# Understanding participation dilemmas in community mobilisation: can collective action theory help?

**DOI:** 10.1136/jech-2018-211045

**Published:** 2018-10-30

**Authors:** Lu Gram, Nayreen Daruwalla, David Osrin

**Affiliations:** 1 Institute for Global Health, University College London, London, UK; 2 SNEHA (Society for Nutrition, Education and Health Action), Mumbai, India

**Keywords:** empowerment pr, health behaviour, health promotion, social epidemiology, social science

## Abstract

Community mobilisation interventions have been used to promote health in many low-income and middle-income settings. They frequently involve collective action to address shared determinants of ill-health, which often requires high levels of participation to be effective. However, the non-excludable nature of benefits produced often generates participation dilemmas: community members have an individual interest in abstaining from collective action and free riding on others’ contributions, but no benefit is produced if nobody participates. For example, marches, rallies or other awareness-raising activities to change entrenched social norms affect the social environment shared by community members whether they participate or not. This creates a temptation to let other community members invest time and effort. Collective action theory provides a rich, principled framework for analysing such participation dilemmas. Over the past 50 years, political scientists, economists, sociologists and psychologists have proposed a plethora of incentive mechanisms to solve participation dilemmas: selective incentives, intrinsic benefits, social incentives, outsize stakes, intermediate goals, interdependency and critical mass theory. We discuss how such incentive mechanisms might be used by global health researchers to produce new questions about how community mobilisation works and conclude with theoretical predictions to be explored in future quantitative or qualitative research.

## Introduction

In *Rules for Radicals*, legendary community organiser Saul Alinsky asserted that efforts to mobilise communities were bound to fail if activists did not appeal to community members’ self-interest.^1^ By ‘self-interest’, Alinsky was not only referring to material or ‘selfish’ interests, but to any motivation that compelled individuals to act.^1^ In contemporary discourse, we might call such a force an incentive. we describe a theoretical framework for conceptualising the mechanisms through which community members may be incentivised to participate in community mobilisation interventions at a grassroots level.

Community participation is an important concern in global health, in which it is enshrined as a right and social good by the 1978 Alma Ata Declaration. Societal and systemic improvement, healthcare provision and disease control are a shared responsibility of policy, governance, public, private and third sector stakeholders. The enactment of this responsibility is influenced by individual and collective efforts, including advocacy and lobbying, which originate in diverse communities. At the grassroots level community participation is argued to facilitate the sustainability of health programmes,^2^ improve fit between programme objectives and local needs^3^ and tackle cultural, societal and environmental barriers to health that are costly or impractical to address through other means.^4 5^

Community mobilisation interventions are frequently initiated to promote community participation.^6^ As they are complex interventions that involve multiple, recursive feedback loops and emergent outcomes, their evaluation has become a feature of global and public health discussion.^7–11^ They can be defined as interventions in which local individuals collaborate with external agents in identifying, prioritising and tackling the causes of ill-health based on values of bottom-up leadership and empowerment.^4 12^

For example, participatory women’s groups have been used in Nepal, India, Malawi and Bangladesh to promote maternal and newborn health.^13^ In these interventions, a trained peer facilitator leads group members through a participatory learning and action cycle in which they prioritise, plan and implement strategies to address local health problems.^14^ A meta-analysis of trials of this approach suggested that it was associated with a 20% population-level reduction in neonatal mortality.^13^ Other applications of community mobilisation include community monitoring of health services,^15^ sex worker collectivisation to promote sexual health,^16^ self-help groups to prevent intimate partner violence^17^ and village health clubs to address hygiene and sanitation.^18^

These interventions typically involve non-specialist community members in grassroots collective action for the benefit of the community. For example, women’s groups in rural Malawi, India and Bangladesh organised health education sessions, community ambulance services, and mobile clinics.^19–22^ Community members in rural Uganda monitored and reported on health worker performance to local health committees.^15^ Members of health and sanitation clubs in rural Zimbabwe dug latrines and garbage pits and conducted home visits to monitor each other’s construction work.^18^ Self-help groups in South Africa conducted a sit-in at a local police station for poor service, established curfews on the sale of liquor & a ban on alcohol sales to adolescents, and coordinated rape prevention meetings between village stakeholders.^23 24^

Whether attending a protest, joining a self-help group or enlisting as a volunteer, a high level of participation in collective action is plausibly required for successful outcomes in many intervention contexts, but participation varies. A meta-analysis of trials of participatory women’s groups to prevent maternal and neonatal mortality found that attendance ranged from 2% to 51% of the target population. It also showed a linear relationship between the proportion of women attending group meetings and population impact, as well as a minimum need for 30% group attendance to produce impacts on mortality.^13^

## Participation dilemmas in community mobilisation

A need for high levels of participation in collective action to produce shared benefits often generates social dilemmas.^25^ In *The Logic of Collective Action*, Mancur Olson pointed out that, when large numbers of participants are required to achieve a shared outcome, the personal impact of a single participant is small relative to the cost of participation.^26^ The implication is that individuals will have an interest in abstaining from participating in collective action and free riding on others’ contributions, even if no benefit is produced if nobody participates. We might call this phenomenon, a ‘participation dilemma’.

For example, a community mobilisation programme to promote sexual health in urban India organised sex workers into collectives which conducted a number of disruptive protests, including rallies, hunger strikes and *gherao* (encirclement of public buildings), to oppose street violence and raise awareness of sex workers’ rights.^27^ Participation in such protests involved considerable risks of violent backlash, yet all resident sex workers benefited, whether or not they participated. It was impossible for participants to exclude non-participants from enjoying greater community awareness of sex workers’ rights: in economic terms, awareness-raising was a non-excludable good.

Collective action in community mobilisation interventions typically produces such non-excludable goods. Rural community members holding health services accountable for deficient performance cannot exclude non-participants from enjoying the benefits of improved service quality.^15^ Self-help groups establishing a curfew on the sale of liquor and a ban on alcohol sales to adolescents cannot exclude non-members from benefiting from reduced risks of alcohol use.^23 24^ Members of health and sanitation clubs seeking to reduce transmission of waterborne disease cannot exclude non-members from enjoying the benefits.^18^ Members of women’s groups lobbying health authorities for staff at mobile health clinics cannot ask for these workers to exclude non-members from their services.^19^ In some contexts, simply attending a women’s group produces non-excludable benefits by challenging deeply held cultural norms mandating women’s physical seclusion and confinement to the home.^28^

In other situations, the direct benefits are excludable, but sharing still occurs out of altruism. For example, members of women’s groups invested considerable time and effort in lobbying donors to pay for bicycle ambulances, monitoring and maintaining bicycle quality and operating shifts to take patients to hospital.^19^ In principle, they could have made a profit on their bicycle ambulance service, but in practice it was provided for free or only charged for at the bare minimum to cover production costs.^19^ Free rider problems still exist in these examples. Altruistic community members may all care about increasing access to emergency transport for residents in need, but each member has an individual temptation to let others do the work in setting up and maintaining the ambulance service. In social psychology, this is related to ‘the bystander effect’,^29^ whereby individuals observing an emergency incident, such as a mugging or a rape, are *less likely* to help the afflicted person, the more bystanders are present who could potentially help.

## Material incentives for participation?

To solve participation dilemmas, global health researchers have grappled with whether to provide material incentives, such as cash benefits for participation.^28^ Community members are often vocal about their desires for benefits from participation and may explicitly attribute lack of commitment to insufficient material incentives.^18 30 31^ Attendance rates at women’s groups in rural Nepal increased from <40% to over 90% when cash or food transfers were provided at group meetings.^28^ Development researchers have also noted how community members suffering from ‘participation fatigue’ have withdrawn from projects after being asked repeatedly to contribute in the absence of personal benefit.^32 33^

Many debates over the use of incentives for community mobilisation mirror arguments over their use in community health worker programmes.^34^ Some have asserted that people in extreme poverty should be provided material incentives to tackle structural vulnerabilities preventing them from participating.^35 36^ Others have contended that the resulting participation is tokenistic, opportunistic and unsustainable, and makes interventions more costly to deliver.^32 37^

Nonetheless, community mobilisation interventions differ critically from community health worker programmes in their emphasis on community members *themselves* identifying, prioritising and owning solutions to local health problems. For example, external agents cannot simply pay community members to operate a bicycle ambulance service from the start as this would undermine the very process of reflection, conception and action thought key to community empowerment.^38^

At most, external agents can facilitate the collectivisation process by supplying trained facilitators to create safe spaces for dialogue and reflection,^4 23^ providing material incentives for attendance^28 39^ or supplying a budget for groups to administer as they see fit.^40^ None of these approaches dispel fundamental dilemmas between self-interest and collective interest in subsequent decisions to participate in collective action to address local health problems.

## Solving participation dilemmas: can collective action theory help?

Collective action theory^41^ provides a rich, principled framework for analysing social dilemmas in collective action. It has been tested in research on political protest,^42^ voting,^43^ union membership^26^ and participation in social movements,^44^ in which it has been extremely influential. It is well-known for its application to environmental politics,^45^ in which it seeks to address Hardin’s^46^ ‘tragedy of the commons’. A large international effort in psychology, economics and social anthropology is also underway using experimental games informed by collective action theory to measure cross-cultural variation in cooperation and conflict.^47 48^ These place participants in artificial dilemmas involving real financial stakes where they have to choose between their own and their collective interest. The same games are used increasingly to measure trust and cooperation in international development projects.^49 50^

As of October 2018, Olson’s *The Logic of Collective Action* had garnered over 40 000 citations on Google Scholar, but it does not seem to have made much of a mark on global health research. While collective action-oriented interventions are frequently deployed and evaluated in global health,^16 17 37 51 52^ existing studies have, to our knowledge, paid less attention to potential conflicts of interest between individual self-preservation and collective interest. A PubMed search on ‘social dilemma’ yielded only two studies concerning a global health issue,^53 54^ while a 2018 review in *the Annual Review of Psychology* on social mobilisation research^55^ only referenced a single study on health behaviour.^56^

At a minimum, therefore, we have an opportunity to consider the use of collective action theory more widely in global health. Of particular interest to health programming are an impressive number of proposed solutions to the free rider problem that have accumulated over the past 50 years; political scientist Lichbach^57^ listed no fewer than 39 potential mechanisms. Global health researchers might usefully employ these to ‘open the black box’^58^ of community mobilisation and pose more nuanced questions about how interventions achieve—or fail to achieve—their aspirations.

Before discussing the range of proposed solutions, we emphasise that we are not making evidenced claims for their efficacy. Each solution is far from perfect and is by no means a recipe for generating collective action. We outline theory, not to provide answers, but to point researchers towards a broader range of questions.

## Solving participation dilemmas: a theoretical framework

Table 1 summarises proposed solutions to participation dilemmas from collective action theory, divided into five general groups.

**Table 1:** Proposed solutions to participation dilemmas

Solution	Explanation	Examples
A. Selective incentives	Tangible rewards for participants, or penalties for non-participants	Stipends for volunteers; free food, training or entertainment for group members; education on ’hook topics' that are unrelated to the primary purpose of a self-help group to attract participants.
B. Social incentives	Incentives generated by social interaction with other community members	Opportunities for building individual social capital, displays of approval of participation or disapproval of non-participation by community members.
C. Outsize stakes, intermediate goals, interdependency	Situations in which the incentive structure does not produce a participation dilemma	A wealthy patron willing to build a clean water supply for the whole village; a health and sanitation club satisfied with raising awareness rather than changing behaviours; a troupe of activist street theatre performers who depend on each other for success.
D. Intrinsic benefits	Psychological or moral rewards for participation or penalties for non-participation	The benefits of being able to express outrage, gain a sense of agency, feel part of a greater cause, feel less lonely, express one’s identity, show solidarity or perform one’s moral duty.
E. Critical mass	An initial group of highly motivated participants sets off a chain reaction that rapidly drives further participation up	A small, initial group of street protesters against police inaction on violence against women successfully convince authorities to take action on a case of domestic violence, thereby persuading other community members to join future protests.

(A) *Selective incentives* are tangible rewards for contributors or penalties for non-contributors, which incentivise participation by making individuals personally lose out if they free ride.^26^ In community mobilisation interventions, participants are often offered stipends for volunteer outreach^35^ or financial training or microcredit loans for joining self-help groups.^36 39^ Literacy classes,^59^ free food,^30^ reimbursement of travel costs^30^ or opportunities for games and entertainment^21^ at group meetings also fall into this category. As community members decide on their own strategies for health promotion in community mobilisation interventions, such incentives cannot be provided for specific health behaviours, but they may still form part of a larger solution by encouraging meeting attendance.

(B) *Social incentives* arise from participants’ interactions with other community members. They often invoke ideas about social norms, reputation and social capital. For example, social norms may lead group or community members to actively display approval of participants and disapproval of non-participants.^60^ Participation may serve as a costly signal for ’good character' and help establish a favourable reputation in the wider community;^61^ this may in turn confer future benefits of trust and cooperation. Participation may also help build relationships with other community members that can be leveraged for future social support, that is, ‘individual social capital’.^62^

Such incentives may be particularly likely to be generated when group members collaborate in the production of ‘club benefits’^57^: benefits that are collectively owned by members, but exclude the community at large. For example, women’s groups sometimes established mutual funds for emergency healthcare, to which members contributed to generate a pool of savings.^63^ Since access to funds was controlled by group members,^19 63^ women probably needed to maintain strong relationships with other members to retain access. At the same time, the funds required frequent group interaction that might produce internal norms of approval for participation and disapproval for free riding. This would in turn strengthen women’s incentive to maintain a reputation for contributing a fair share to group work.

(C) *Outsize stakes, intermediate goals and interdependent groups* are means by which the need for collective action may not involve a participation dilemma. Some individuals may have such an outsize stake in addressing a particular public issue that they are willing to either bear the full cost of supplying the non-excludable good or contribute to it despite their personal impact being small relative to personal cost.^64^ For example, a wealthy patron might be willing to build a clean water supply for his whole village, or a politically well-connected community member may be able to lobby for policy change on behalf of the community. Individuals may also see sufficient value in realising easily achievable intermediate goals that the achievement of the final goal is less important.^64^ For example, a health and sanitation club may be satisfied with raising awareness rather than changing behaviours in the short-term. Collective actions involving small, interdependent groups also pose less of a participation dilemma. When each individual contribution is essential to the end result, a single free rider would compromise the whole enterprise.^26^ For example, a small troupe of activist street theatre performers may depend intimately on each other for success. In general, when personal contributions make large, unique impacts, social dilemmas pose less of a problem.

(D) *Intrinsic benefits* or ‘expressive’ or ‘symbolic’ benefits^65^ are psychological or moral benefits that are intrinsic to the act of participating, unlike ‘instrumental benefits’ that accrue as a result of participating. All the above motivations (A–C) were oriented towards obtaining instrumental benefits. Intrinsic benefits include gaining a sense of agency, feeling part of a greater cause, expressing outrage, feeling the ‘warm glow’ of helping others, feeling less lonely, expressing one’s identity, showing solidarity or performing one’s moral duty.^66^ Some political scientists have asserted that intrinsic benefits are the only rational incentive for participation in voting given the vanishing likelihood of any single vote changing the outcome of an election.^65^ Many intrinsic benefits depend on individual perceptions of community issues; thus, skilled communication and ‘framing’^67^ of issues is crucial for activating them as motivations for collective action. For example, feminist microcredit non-government organisations in rural India successfully coordinated a number of large-scale protests against witch-hunts among tribal women by framing them as part of their struggle for independence from abusive husbands and exploitative employers.^68^

(E) *Critical mass* theory^69^ or ‘tipping point theory’^70^ involves a two-stage mechanism for solving participation dilemmas. First, highly motivated individuals who are willing to participate when few others have joined provide an initial critical mass. They may be incentivised by any of the mechanisms discussed above. Next, ‘bandwagon effects’ may induce further participation and ideally set up a positive feedback loop that drives participation up.

Bandwagon effects may come from three sources. First, as more people join, individual costs and rewards may become more favourable.^69^ For example, attending a peer support group for people living with HIV may be deeply stigmatising for the first attendant, but increasingly socially acceptable as the group becomes bigger and more widely known. Second, the potential personal impact on collective success may increase.^64^ The first 20 people who rally against female genital cutting in a village may be dismissed as outliers, but the next 20 who attend may be enough to worry community members that popular opinion is shifting. Third, individual participation may provide social proof to other community members of the value of participating.^71^ Street protesters against police inaction on violence against women may persuade others to join future protests if they successfully convince authorities to take action on a specific case of violent abuse.

## Towards a future research agenda

Collective action theory raises many specific questions about the drivers of participation.

*Does participation encourage or discourage future participation?* Are individuals encouraged or discouraged from participating themselves if they see others participate? If social incentives (scenario B) or bandwagon effects (scenario E) predominate, we might expect individuals to be encouraged by learning of others’ participation. Indeed, social psychologists have often asserted that information campaigns should avoid highlighting gaps in collective action on social issues, lest they inadvertently establish non-participation as a descriptive norm in community members’ minds.^72^ For example, an intervention providing households with information on their own and their neighbours’ electricity consumption to discourage electricity waste saw below-average households *increase* their consumption levels to match the descriptive norm.^73^

However, if individuals primarily participate to supply a non-excludable good (scenario C), we might expect them to be discouraged by widespread participation because opportunities to free ride become greater. For example, one study providing party activists for a major European election with information about the canvassing intentions of their peers saw activists *reducing* their activity levels after learning that peers engaged in more canvassing than previously thought.^74^

Finally, if participation is motivated by selective incentives (scenario A) or intrinsic benefits (scenario D), the expected number of participants—everything else being equal—should have no effect on people’s decision to participate. Participation in collective action may not necessarily encourage future participation, but may either encourage or discourage it depending on the underlying incentive mechanism.

*Does demand for the non-excludable good matter?* Folk wisdom holds that the greater the demand for a common good, the more likely it is that people will try to supply it. Marx famously theorised that oppressed people do not rise up against their oppressors because ‘false consciousness’ renders them unable to recognise the daily injustices they are subjected to.^75^ Brazilian activist Paulo Freire^76^ prescribed group dialogue and reflection to foster a ‘critical consciousness’ among oppressed peoples to inspire collective action against such injustices.

However, collective action theorists have long questioned the obviousness of a link between shared grievances and participation in collective action.^44^ A 1983 Gallup poll showed that 40% of people in the USA believed it was likely that there would be nuclear war by 1998, and 70% believed they would not survive it, but only a very small fraction of citizens actively protested the proliferation of nuclear missiles in the 1980s.^77^

Our theoretical framework predicts that demand for a non-excludable good does not always affect participation rates. Participation motivated by selective incentives, social incentives or bandwagon effects (scenarios A, B and E) should be little affected because these incentives can exist even without demand for the non-excludable good. Participation motivated by intrinsic benefits (scenario D), on the other hand, probably increases with perceived need for the good as stronger grievances generate greater moral outrage or sense of duty. Evidently, participation motivated primarily by the desire to supply the non-excludable good (scenario C) increases with demand for it.

*How are second-order participation dilemmas resolved?* First-order participation dilemmas are social dilemmas concerning the production of a non-excludable good: improved community hygiene and sanitation, community awareness about women’s rights, improved quality of health services, legislation and policy change. Second-order participation dilemmas are dilemmas concerning the production of social incentives to encourage participation in first-order collective action.

For example, community-led solutions to promote sanitation and hygiene frequently involve social sanctions or monetary fines for open defecation.^78^ While the need to encourage large numbers of community members to stop defecating in the open presents a first-order participation dilemma, the need to persuade the same community members to sanction other residents for defecating in the open presents a second-order participation dilemma.

Each community member seeing their neighbour defecate in the open would have to admonish them repeatedly to change an ingrained habit. Similarly, fining a neighbour for defecating in the open requires a great deal of personal effort in involving the relevant authorities and carries personal risks of backlash. Even so, a single neighbour’s change in behaviour would have a negligible effect on the community’s overall risk of infectious disease—the costs of enforcing this social norm may outweigh its personal rewards. This creates a second-order participation dilemma in which each member is individually tempted to ignore instances of open defecation despite a collective interest in establishing a health-promoting social norm.

While global health researchers routinely invoke social incentives to explain participation in collective action,^31 79^ such explanations often assume away hidden second-order participation dilemmas. Understanding how second-order participation dilemmas are resolved may be useful for unlocking the potential of social incentives as a strategy for behaviour change.

*Are some cultures better able to promote collective action than others?* One of the most enduring theoretical constructs in cross-cultural psychology has been the distinction between individualist and collectivist cultures.^80^ Individuals from collectivist cultures are thought to define themselves as part of a larger, interdependent group and prioritise collective rather than individual goals.^80^ Non-Western cultures are often considered more collectivist than Western cultures. Reviewing the marked differences in values, preferences and self-conceptions between cultures reported in existing literature, Henrich *et al* 81 asked rhetorically whether standard Western samples of participants in mainstream psychology were some of the ‘weirdest people in the world?’

However, the direction of cultural difference does not consistently point towards greater cooperation in collectivist cultures. While survey data from many non-Western cultures indicate more interdependent self-concepts and lower preferences for autonomy, data from experimental games involving real financial stakes indicate substantially *greater* conformity with economic models of rational self-interest maximisation.^81^ At the same time, forms of anticooperation have emerged in samples from societies such as Turkey, Korea and Oman, traditionally regarded as collectivist, where participants exact financial punishment on people who contribute to collective outcomes.^81^

Our own theoretical framework does not unambiguously predict greater capacity for collective action in collectivist cultures. If collectivist individuals place greater value on collective outcomes, demand for the collective good will increase, but—as noted above —this does not always translate into higher levels of participation. If internalised cultural values create intrinsic benefits to collective action (scenario D), social norms mandate informal social rewards or sanctions (scenario B), or (quasi-)legal institutions administer tangible rewards or sanctions (scenario A), we might expect enhanced capacities for collective action, but only if these incentives outweigh any contrary incentives that deter participation.

## Limitations

Concerns with empowerment and community-building are better served by normative rather than positive theories of participation.^82 83^ Many group processes fit awkwardly into a cost-benefit framework such as in-group/out-group categorisation,^84^ learning and socialisation in groups,^85^ collective cognitive biases^86 87^ and group emotion.^88^ Non-participation may arise for reasons other than free riding^43^: individuals may have no stake in a collective outcome or even be opposed to it. We do not advocate displacing the above concerns with a narrow focus on participation dilemmas.

## Conclusion

Community mobilisation has been successfully used to improve health in many low- and middle-income contexts,^13 15–18^ but our understanding of the mechanisms through which it achieves its outcomes is evolving. In this paper, we suggested a theoretical framework for conceptualising the mechanisms through which participation in community mobilisation interventions happens. We see our framework as a pragmatic basis from which to pose more nuanced questions and help open the black box of community mobilisation. Collective action theorists tend to believe that large, poorly organised constituencies are routinely outmanoeuvred by smaller, better organised groups.^89^ Although community members rightly play a central role in the implementation of community mobilisation interventions, we cannot expect individuals taking volunteer time from their overburdened lives to have all the answers to the manifold problems of mobilising, organising and delivering effective action. Heeding Alinsky’s call to think through community members’ motives for participation might move us one step closer towards this end.^1^

**What is already known on this subject**

- Community mobilisation is a complex social intervention producing emergent and often unpredictable outcomes.
- Community mobilisation often seeks to engender grassroots collective action to address shared determinants of ill-health, poverty and powerlessness.
- Grassroots collective action often requires high levels of participation to be effective.

**What this study adds**

- Collective action theory is well-known in the social sciences, but has been less explored in global and public health.
- Collective action theory provides a theoretical framework for maximising participation and minimising free riding in collective action.
- Collective action theory may enable a better understanding of how community mobilisation interventions achieve high levels of participation.
